# Provocation of brachial plexus and systemic symptoms during the elevated arm stress test in individuals with myalgic encephalomyelitis/chronic fatigue syndrome or idiopathic chronic fatigue

**DOI:** 10.1186/s12967-025-06137-7

**Published:** 2025-01-22

**Authors:** Charles C. Edwards, Julia M. Byrnes, Camille A. Broussard, Alba M. Azola, Meghan E. Swope, Colleen L. Marden, Renee L. Swope, Ying Wei Lum, Richard L. Violand, Peter C. Rowe

**Affiliations:** 1 Division of Adolescent and Young Adult Medicine, Departments of Pediatrics, Johns Hokins University School of Medicine, 200 N. Wolfe St., Room 2077, Baltimore, MD 21287 USA; 2 Division of Adolescent and Young Adult Medicine, Departments of Pediatrics, Department of Physical Medicine and Rehabilitation, Johns Hokins University School of Medicine, 200 N. Wolfe St., Room 2077, Baltimore, MD 21287 USA; 3 Division of Vascular Surgery, Department of Surgery, Johns Hopkins University School of Medicine, 600 North Wolfe Street, Halsted 668, Baltimore, MD 21287 USA

**Keywords:** Myalgic encephalomyelitis/chronic fatigue syndrome, Thoracic outlet syndrome, Elevated arm stress test, Joint hypermobility, Ehlers-Danlos syndrome, Orthostatic intolerance

## Abstract

**Background:**

We have noted that some adolescents and young adults with myalgic encephalomyelitis/chronic fatigue syndrome (ME/CFS) report difficulty with arms-overhead activities, suggestive of brachial plexus dysfunction or thoracic outlet syndrome (TOS). In the TOS literature, diagnostic maneuvers focus on the provocation of upper limb symptoms (arm fatigue and heaviness, paresthesias, neck and upper back pain), but not on elicitation of systemic symptoms.

**Objectives:**

To estimate the proportion of patients with fatiguing illness who experience local and systemic symptoms during a common maneuver used in evaluating TOS—the elevated arm stress test (EAST).

**Methods:**

Patients were eligible for this retrospective study if they had been referred to the Johns Hopkins Chronic Fatigue Clinic between January 2020 and July 2023 and (a) reported difficulty maintaining arms-overhead postures, (b) were evaluated with an abbreviated one-minute EAST, and (c) had not undergone surgery in the upper limb, neck, or skull base. Modified EAST procedure: patients sat with their arms in a “hands up” or “candlestick” position while opening and closing their hands every 2–3 s repeatedly for 1 min, rather than the customary 3 min. The test was considered abnormal for local symptoms if the participant experienced pain, fatigue, heaviness, paresthesias, warmth or tremulousness in the upper limb, shoulder, neck, head, or upper back. The test was considered abnormal for systemic symptoms if the participant experienced overall fatigue, cognitive fogginess, lightheadedness, racing heart, diaphoresis, dyspnea, overall warmth, or nausea.

**Results:**

Of 154 patients evaluated during the study period, 64 (42%) met the eligibility criteria (61/64 female, median age 18 years [range, 13 to 50]). Of the 64, 50 (78%) had ME/CFS, 13 (20%) had idiopathic chronic fatigue with associated orthostatic intolerance (OI), and one had idiopathic chronic fatigue without OI. Of the 64, 58% had evidence of joint hypermobility. Local symptoms were provoked by EAST in 62/64 (97%) within a median of 20 s. During EAST, 26/64 (41%) reported systemic symptoms (1 had only systemic but no upper limb symptoms), most commonly lightheadedness (19%) and generalized fatigue (11%).

**Conclusions:**

Even with an abbreviated test duration, the EAST maneuver provoked local and systemic symptoms in a substantial proportion of patients with chronic fatigue, OI, and ME/CFS who had reported difficulty with arms-overhead postures. Further studies are needed to explore the prevalence of brachial plexus or TOS symptoms in unselected individuals with ME/CFS or OI, and the proportion with systemic symptoms during and after EAST.

## Background

As defined by the United States Institute of Medicine (IOM) in 2015, myalgic encephalomyelitis/chronic fatigue syndrome (ME/CFS) is a complex, multisystem disorder characterized by a substantial impairment in overall function, usually associated with profound fatigue [1]. Other core symptoms include unrefreshing sleep, post-exertional malaise (PEM, a worsening of fatigue as well as other symptoms following physical or cognitive activity, orthostatic stress [2], or neuromuscular strain [3]), and either orthostatic intolerance or cognitive dysfunction [1]. A risk factor for both ME/CFS and orthostatic intolerance is connective tissue laxity, as evidenced by joint hypermobility or Ehlers-Danlos syndrome [4–8]. The prevalence of ME/CFS is increasing, as many patients with substantial impairment following SARS-CoV-2 infection (long COVID) meet criteria for the diagnosis [9, 10]. Up to 1.3% of the US adult population had been diagnosed with ME/CFS in 2022; the proportion with ME/CFS who have not received a formal diagnosis is likely higher [11]. Improved understanding of the pathogenesis of ME/CFS symptoms and more targeted treatments are needed to decrease the burden of this chronic illness.

Our previous research has shown that, compared to healthy controls, individuals with ME/CFS have a significantly higher prevalence of restrictions in symptom-free range of motion (ROM) of the limbs and spine [12–14]. More recently, we have noted that some adolescents and young adults with ME/CFS report difficulty with arms-overhead activities, suggestive of brachial plexus dysfunction or thoracic outlet syndrome (TOS). The thoracic outlet is a region of the body that extends from the lateral neck into the anterior chest. Found within this region are the brachial plexus and the large blood vessels traveling with it. TOS is a condition wherein multiple and disparate symptoms may occur due to compression or irritation of brachial plexus components or compression of the arteries or veins [15–19]. Neurogenic TOS or nTOS is the descriptor applied when symptoms arise from the brachial plexus. Involvement of the vessels accompanying the brachial plexus may lead to arterial TOS or venous TOS. Neurogenic TOS commonly results from trauma, tissue abnormalities, and repetitive use [16]. Entrapment sites for neural strain and compression that can give rise to symptoms of nTOS are at the scalene triangle, costoclavicular space, and beneath the pectoralis minor. Neurogenic TOS occurs far more often than vascular TOS and accounts for 95% of cases [16]. The Society for Vascular Surgery recommends both the upper limb tension test (ULNT1) and the elevated arm stress test (EAST, also known as the Roos test) as examination maneuvers in suspected nTOS. [19–21]

In the TOS literature, diagnostic maneuvers focus on the provocation of upper limb symptoms (arm fatigue and heaviness, paresthesias, neck and upper back pain), but not on elicitation of systemic symptoms. We are not aware of any reports focusing on TOS in the ME/CFS literature. The current study describes the onset and timing of local and systemic symptoms during a common TOS diagnostic maneuver, the elevated arm stress test (EAST) in selected individuals with ME/CFS and other fatiguing conditions who report difficulty with arms-overhead or arm-extended postures. Given the claims that there is an association of TOS with joint hypermobility and Ehlers Danlos syndrome [22, 23], we examined the prevalence of elevated Beighton scores in this population and whether this influenced symptom provocation during the EAST.

## Methods

### Participants

This was a retrospective chart review. Patients were eligible for this study if they had been referred to the Johns Hopkins Chronic Fatigue Clinic between January 2020 and July 2023, and (a) they reported difficulty maintaining arms-overhead or arms-extended postures, and (b) their clinical examination had included an abbreviated one-minute EAST (see below). We excluded those who had undergone surgery in the upper limb, neck, or skull base, as this could have affected the symptoms reported during the EAST.

### Diagnostic criteria

ME/CFS: We classified individuals as having ME/CFS if they satisfied the 2015 IOM definition [1], which required the following core symptoms: (1) a substantial impairment in the ability to engage in pre-illness levels of occupational, educational, social, or personal activities, persisting for more than 6 months, accompanied by profound, novel fatigue that is not the result of ongoing excessive exertion and is not substantially alleviated by rest, (2) post-exertional malaise, (3) unrefreshing sleep, and either (4a) cognitive impairment or (4b) orthostatic intolerance. We defined idiopathic chronic fatigue as fatigue that lasted at least 6 months but did not meet the IOM criteria.

Orthostatic Intolerance: We classified patients as having orthostatic intolerance if they had had frequent orthostatic symptoms in the absence of hemodynamic abnormalities, or if they had been diagnosed with neurally mediated hypotension (NMH) or postural tachycardia syndrome (POTS) using either a head-up tilt table test or a passive standing test. The diagnosis of NMH required a sudden and sustained drop in systolic blood pressure of at least 25 mm Hg compared to the supine values, with no associated increase in heart rate, and accompanied by symptoms of presyncope (severe lightheadedness, weakness, nausea, or diaphoresis) [24]. The diagnosis of POTS for those 12–19 years required at least a 40-beat increase in heart rate compared to supine values over 10 min of standing or head-up tilt testing, with chronic orthostatic symptoms, and without orthostatic hypotension within the first 3 min. For those 20 years and older, a 30-beat increase was sufficient for the diagnosis. [25]

Joint Hypermobility/Ehlers-Danlos syndrome: At the initial visit in all patients referred to the Chronic Fatigue Clinic, we routinely obtain a 9-point Beighton score to screen for joint hypermobility, using a goniometer for measuring joint angles [26]. For the Beighton score, an examiner assigns one point on each side of the body for (a) passive dorsiflexion of the 5th finger at the metacarpophalangeal joint of > 90 degrees, (b) passive apposition of the thumb to the flexor aspect of the forearm, (c) extension of the elbow > 190 degrees, (d) extension of the knee > 190 degrees. The 9th point is for the ability to place the palms flat on the floor while bending over at the waist without bending the knees. In previous work, we had used the Beighton cut-off score of ≥ 4 to indicate joint hypermobility, and used the same cut-off in this study as well [5]. By definition, individuals diagnosed with Ehlers-Danlos syndrome were included as having joint hypermobility. We did not perform genetic testing to confirm the diagnosis of Ehlers-Danlos syndrome (EDS), as there is no known genetic mutation for the most common type of EDS (hypermobile EDS).

### Study procedures

Modified EAST procedure: Patients were seated in a chair, with their feet on the floor, back supported, and arms in a “hands up” or “candlestick” position (meaning the arms were abducted to a 90-degree angle in the frontal plane, with forearms vertical and palms forward). Patients were instructed to then open and close their hands every 2–3 s repeatedly for 1 min (rather than the customary 3 min). The 1-min duration has been used by others [27], and was chosen to reduce the likelihood of provoking prolonged ME/CFS symptoms following the test.

Patients were asked to report to the examiner as soon as they experienced the onset of any symptom in the upper limbs or any symptom in general. The test was considered abnormal for local symptoms if the participant experienced pain, fatigue, heaviness, paresthesias, warmth or tremulousness in the upper limb, shoulder, neck, head or upper back. The test was considered abnormal for systemic symptoms if the participant experienced overall fatigue, cognitive fogginess, lightheadedness, racing heart, diaphoresis, dyspnea, overall warmth, and nausea. We calculated the overall prevalence of abnormal EAST tests as those with either an abnormal local or an abnormal systemic test. Patients were permitted to stop the test at their discretion if their symptoms exceeded their own level of comfort.

Upper limb neurodynamic test 1 (ULNT1): Based on earlier experience, we routinely perform an upper limb neurodynamic test 1 (ULNT1, often termed the upper limb tension test), in accordance with the method described by Butler [28]. With the patient positioned supine and facing forward, arms at the sides and with legs together and knees extended, the examiner then performs the following sequence of maneuvers: a) abduction of one of the subject’s arms to 110 degrees while the shoulder girdle is held in a depressed, but neutral position of glenohumeral rotation, with the elbow flexed to 90 degrees, b) supination of the forearm with wrist, thumb, and finger extension, c), lateral rotation of the arm to 90 degrees, d) elbow extension to the point at which the subject reports stretch along the upper limb, e) contralateral neck side bend to bring the ear close to the contralateral shoulder, with the head facing forward (looking up at the ceiling), f) ipsilateral neck side bend toward the shoulder of the limb being tested, again with the head facing forward. Full elbow extension is measured as 180 degrees. An abnormal range of motion on this test consists of an elbow angle less than 170 degrees at the onset of stretch in one or both arms. An abnormal symptomatic response consists of provocation of symptoms other than stretch in the anterior shoulder or antecubital fossa or paresthesias in the hand (e.g., discomfort in the back, neck, or head, or lightheadedness).

### Statistical analysis

We used descriptive statistics, including mean (SD) or median and interquartile range (IQR) where appropriate. We calculated the time until the onset of local upper limb as well as systemic symptoms using survival curves. To explore the influence of joint hypermobility on the provocation of symptoms, we compared the survival curves in hypermobile versus non-hypermobile patients using the log rank (Mantel-Cox) test (GraphPad Prism, version 10.3; GraphPad Software, La Jolla, California, USA; www.graphpad.com).

## Results

### Participants

Of 154 new patients evaluated during the study period, we identified 64 (42%) who reported difficulty with arms-overhead or arms-extended positions and had undergone an EAST. The demographic and clinical characteristics of this group of 64 are presented in Table 1. The median age was 18 years, with 46 (72%) ≤ 21 years, 13 (20%) aged 22–30 years, and five (8%) aged 31–50 years.

**Table 1:** Demographic and clinical characteristics of the study sample (N = 64)

Demographic	
Age in years at study entry, median (IQR)	18 (17–22)
Female sex (%)	95%
Race	
White	91%
African American	6%
Asian/ Pacific Islander	3%
American Indian/Alaskan Native	0%
Hispanic	6%
Clinical	
ME/CFS (N = 50)	78%
Idiopathic CF, OI positive (N = 13)	20%
Idiopathic CF, OI negative (N = 1)	2%
Beighton score, median (IQR)	4 (2–6)
Diagnosed EDS or Beighton score ≥ 4	58%

ME/CFS, myalgic encephalomyelitis/chronic fatigue syndrome; CF, idiopathic chronic fatigue; OI, orthostatic intolerance; IQR, inter-quartile range; EDS, Ehlers-Danlos syndrome

All but one participant had orthostatic intolerance as a contributor to ME/CFS or idiopathic chronic fatigue. Seventeen had orthostatic intolerance symptoms in the absence of hemodynamic changes, and 46 met criteria for POTS (one of whom also had NMH). Joint hypermobility as defined by a Beighton score of at least 4 was present in 58%; 27/64 (42%) had been diagnosed with the hypermobile form of Ehlers-Danlos syndrome.

### EAST results

As displayed in Table 2, at least one local symptom was provoked during the EAST in 62/64 (97%; 95% CI 89–99%), the most common of which were upper limb or shoulder fatigue, pain, paresthesias, and heaviness. At least one systemic symptom was reported by 26/64 (41%; 95% CI 29–53%), the most common of which were lightheadedness and overall fatigue (Table 2). One individual had systemic but no local symptoms during the test, and one individual was completely asymptomatic. Thus, combining those with at least one local or one systemic symptom, 63/64 (98%; 95% CI 92–100%) had an abnormal test result.

**Table 2:** Local and systemic symptom responses to the 60-s EAST (n = 64)

**Any upper limb symptom (N = 62)**	**97%**
Upper limb/shoulder fatigue (n = 43)	67%
Upper limb/shoulder pain or discomfort (n = 33)	52%
Upper limb paresthesias (n = 24)	38%
Upper limb heaviness (n = 20)	31%
Upper back/neck pain or discomfort (n = 7)	11%
Upper limb warmth (n = 5)	8%
Upper limb tremulousness (n = 4)	6%
Headaches (n = 3)	5%
Chest tightness (n = 1)	2%
Upper limb coldness (n = 1)	2%
**Any systemic symptom (N = 26)**	**41%**
Lightheadedness (n = 12)	19%
Overall fatigue (n = 7)	11%
Racing heart (n = 4)	6%
Cognitive fogginess (n = 3)	5%
Diaphoresis (n = 3)	5%
Shortness of breath (n = 3)	5%
Overall warmth (n = 2)	2%
Nausea (n = 1)	2%

*EAST* elevated arm stress test

**Fig. 1:**
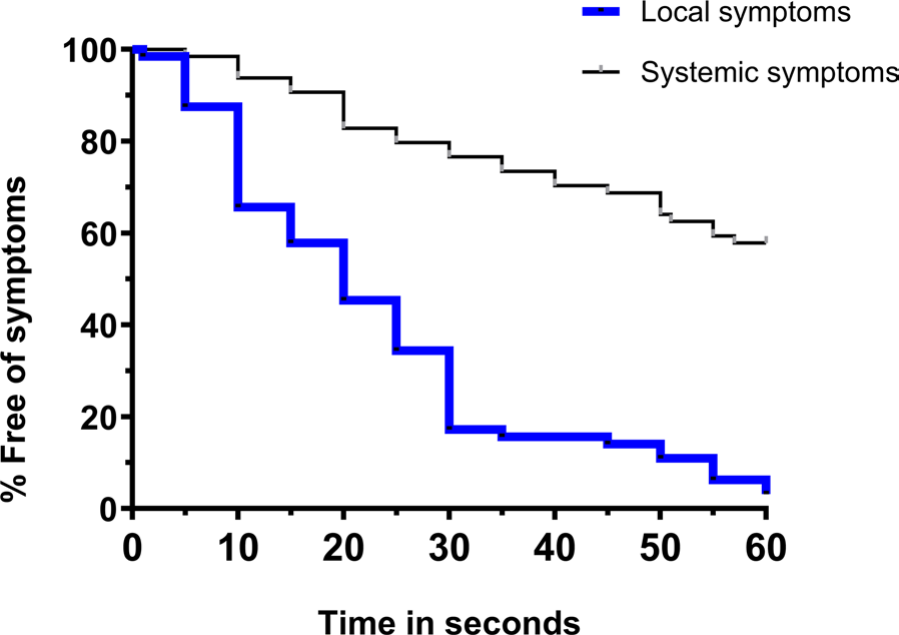
Survival curves of the time in seconds until the onset of local or systemic symptoms during a 1-min elevated arm stress test

Figure 1 displays the lifetable analysis of the time until the onset of local symptoms in the entire group of 64. The median time until the first local symptom was 20 s. The median time until the onset of systemic symptoms could not be calculated as 50% of the participants did not experience a systemic symptom. Among the 41% who reported systemic symptoms, the median onset occurred at 30 s.

**Fig. 2:**
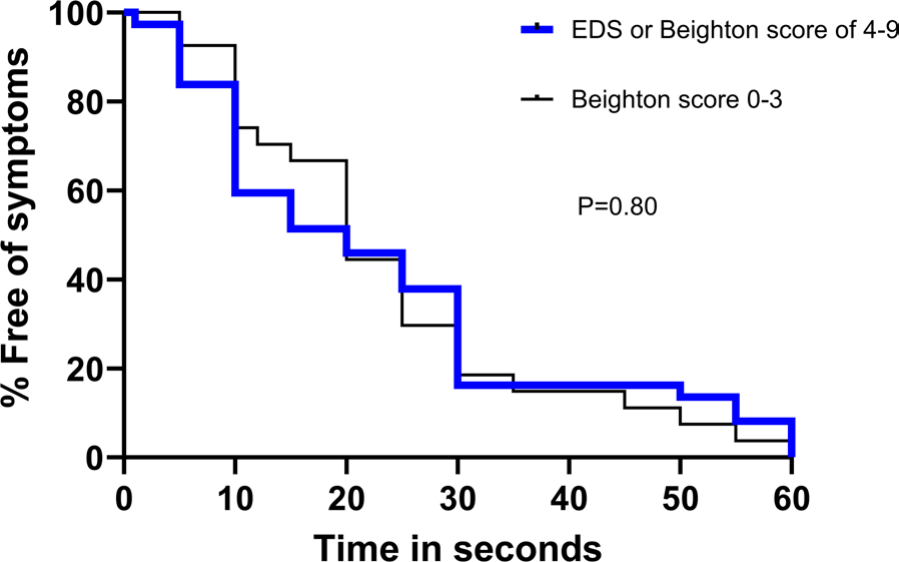
Comparison of the survival curves of the time in seconds until the onset of local symptoms for those with Beighton scores of 0–3 versus 4–9 (P = 0.80, log rank test)

We explored whether there was a difference in the time until the onset of local symptoms between those with and without joint hypermobility. The lifetable analysis showed closely overlapping survival curves (Fig. 2), with a median time to first local symptom of 20 s for both groups (P = 0.80, log rank test).

Sixty-three patients had both an EAST and ULNT1 on the same day. The EAST was more likely to be abnormal than was the ULNT1 (98% versus 43%). Among those with an abnormal test, the median elbow extension was 145 degrees (range 90–160; IQR 140–160). Symptom provocation on this test was not recorded in a systematic manner in the charts.

## Discussion

This study reports several new findings. First, of 154 individuals referred for evaluation of chronic fatigue and orthostatic intolerance, 64 reported difficulty with arms-overhead postures, 98% of whom developed symptoms during an abbreviated 1-min EAST. Second, the EAST provoked systemic symptoms (including lightheadedness, overall fatigue, racing heart, cognitive fogginess, and nausea) in 41% of the 64 individuals. While provocation of local symptoms during EAST has been well described in the literature on thoracic outlet syndrome [15–21], the provocation of systemic symptoms has received little attention. These findings have several implications for clinical care. Treatment of brachial plexus dysfunction is available—ranging from physical therapy to Botox injections and ultimately to surgical procedures such as scalenectomy and partial first rib resection [29]. Recognition of this problem introduces another avenue for treatment of those with ME/CFS and the related conditions we have studied. Our findings suggest that more concerted efforts to ascertain for brachial plexus dysfunction and thoracic outlet syndrome are warranted in this population, as are outcome studies examining the response to treatment.

Another intriguing observation from this study is that the EAST was abnormal in 98% of those reporting symptoms with arms-overhead or arms-extended postures. This prevalence compares to 43% who had an abnormal ULNT1, another commonly used screening maneuver for thoracic outlet syndrome. While neither test is diagnostic for TOS, the ULNT1 has been regarded as the best initial provocative test to screen for TOS [30]. In our sample, it is unclear whether the provocation of systemic symptoms and the higher sensitivity of the EAST are due to some other aspect of the overlapping illnesses included in this study population (idiopathic chronic fatigue, orthostatic intolerance, joint hypermobility, or ME/CFS). It is possible that the EAST involves a sufficient amount of exertion in those with ME/CFS that it provokes general symptoms of the illness. In future studies, it would be important to enroll a comparison group with brachial plexus dysfunction but without ME/CFS to measure the prevalence of systemic symptoms. Given the potential for a variety of physiologic stressors to trigger post-exertional symptom exacerbation in those with ME/CFS (including the 2-day cardiopulmonary exercise test, head-up tilt test, and biomechanical strain using a straight leg raise maneuver), it is possible that travel to the clinic or other examination maneuvers could have lowered the threshold for patients to experience symptoms with the EAST. Once a neurally sensitive area has been identified, little additional stimulus might be required to elicit equivalent responses.

In our study, 98% of the participants had some form of orthostatic intolerance, a core feature of ME/CFS, found in up to 90% of adults and up to 100% of adolescents with ME/CFS [1, 31, 32]. There is limited literature on cardiovascular abnormalities associated with TOS. Gockel and colleagues noted a significantly higher resting mean heart rate in women with TOS compared to female controls (80.5 vs 63.8; P < 0.01). More individuals with TOS than controls had increased sympathetic activity after 3 min of standing (55% vs 11%; P < 0.05) [33]. Kaymak and colleagues reported a patient with tachycardia during EAST testing that resolved following surgical management of the TOS [34]. The same group also described two additional patients with episodic tachycardia when the arms were elevated [35]. From a large group of 1142 individuals with POTS, Kumar and colleagues identified 40 with symptoms suggestive of TOS, half of whom were confirmed to have a diagnosis of vascular TOS [23]. Of the 20 with vascular TOS, 14 had a diagnosis of Ehlers-Danlos syndrome. These authors did not ascertain for neurogenic TOS. While these studies have shown that TOS can be associated with increased heart rate, we are not aware of studies that have examined the potential for TOS and related neural dysfunctions to aggravate ME/CFS symptoms. Neural connections between the intrathoracic nerves and the second and third thoracic sympathetic ganglia, and between the stellate ganglion and the brachial plexus may explain how biomechanical strain in the brachial plexus could translate into changes in heart rate. [36]

Our study had several limitations. Ours was a convenience sample, not a systematic sample of patients with chronic fatigue or ME/CFS. Because of the potential for referral bias, ours may not be representative of all those with ME/CFS at the ages we included. We observed a higher than expected prevalence of females in the study population, which may reflect either the known higher prevalence of joint hypermobility in females (joint hypermobility being a postulated biological risk factor for developing TOS), or a bias in our sample. It will be important in future studies to determine whether males and individuals from non-white populations have similar findings during EAST. Although our sample included 18 adults over the age of 21, the median age of the study participants was 18, and it remains to be seen whether patients in different decades have different rates of abnormal EAST. We did not ascertain for brachial plexus dysfunction using standardized or validated questionnaires such as the Cervical Brachial Symptom Questionnaire [37] or the Disabilities of the Arm Shoulder and Hand (DASH) questionnaire [38]. These instruments would be important to employ in future studies. If individuals with ME/CFS have low scores on the questionnaires, but abnormal responses on the EAST, this would suggest that the EAST results are less due to true brachial plexus dysfunction and more related to the abnormal symptomatic response to exercise in this population. We did not perform an EAST on all individuals, only those reporting difficulty with arms elevated or extended. Our results therefore should not be considered accurate estimates of the prevalence of abnormal EAST in an unselected population of individuals with idiopathic chronic fatigue or ME/CFS. We did not confirm the diagnosis of TOS using Doppler ultrasounds, so we are only able to report that patients had evidence of brachial plexus dysfunction, not confirmed TOS.

## Conclusions

This study sheds light on the potential of the elevated arm stress test (EAST) to elicit both upper limb and systemic symptoms in individuals with chronic fatiguing illnesses, including ME/CFS. Even with an abbreviated test duration, the EAST maneuver provoked systemic symptoms in 41% of those with OI or ME/CFS, a finding previously not explored in the TOS literature. These results provide a rationale for more systematic study of the prevalence of brachial plexus dysfunction in those with ME/CFS and OI using questionnaires, provocative diagnostic maneuvers, and Doppler studies. The association of joint hypermobility and Ehlers Danlos syndrome with brachial plexus dysfunction and TOS also deserves further attention. Early detection of brachial plexus dysfunction has the potential to provide opportunities for tailored treatments, including interventions that have previously shown promise for TOS-related conditions.

## Data Availability

The date applicable to this article are available from the corresponding author upon request.
